# Subcellular Localization of the Sigma-1 Receptor in Retinal Neurons — an Electron Microscopy Study

**DOI:** 10.1038/srep10689

**Published:** 2015-06-02

**Authors:** Timur A. Mavlyutov, Miles Epstein, Lian-Wang Guo

**Affiliations:** 1 Department of Surgery, University of Wisconsin School of Medicine and Public Health, 5151 Wisconsin Institute for Medical Research, 1111 Highland Ave, Madison, WI 53705, USA; 2 McPherson Eye Research Institute, University of Wisconsin School of Medicine and Public Health, 5151 Wisconsin Institute for Medical Research, 1111 Highland Ave, Madison, WI 53705, USA; 3 Department of Neuroscience, University of Wisconsin School of Medicine and Public Health, 41 Bardeen Medical Laboratory, 470 N Charter Street, Madison, WI 53706, USA

## Abstract

The Sigma-1 receptor (S1R) is known to play a protective role in the central nervous system including the retina. A major barrier for understanding the underlying mechanism is an ambiguity of S1R subcellular localizations. We thus conducted the first electron microscopy (EM) study of S1R subcellular distribution in the mouse retina. Immuno-EM imaging showed previously under-appreciated S1R presence in photoreceptor cells. Unlike in other cell types in previous reports, in photoreceptor cells S1R was found in the nuclear envelope but not localized in the endoplasmic reticulum (ER), raising a possibility of S1R-mediated modulatory mechanisms different than conventionally thought. While in bipolar cells S1R was detected only in the nuclear envelope, in ganglion cells S1R was identified predominantly in the nuclear envelope and found in the ER as well. A predominant localization of S1R in the nuclear envelope in all three retinal neurons implicates a potential role of S1R in modulating nuclear activities. Moreover, its absence in the plasma membrane and presence in the subsurface ER cisternae that are juxtaposed to the plasma membrane in ganglion cells may lend mechanistic insights generally important for frequently reported S1R modulations of ion channels in neurons.

Once considered “orphan” receptors without defined biological functions, Sigma receptors are now attracting tremendous interest from diverse research areas, in particular, the central nervous system. Sigma binding sites are classified into Sigma-1 (S1R) and Sigma-2 receptors (S2R). The S1R is unique in that while its amino acid sequence is highly conserved across rodents and humans (>90% identity) it shares no significant homology (<30%) with any other mammalian or non-mammalian proteins^1^. Moreover, recent discoveries reveal that S1R is a unique ligand-operated chaperone that is pro-survival under cellular stress^2^,^3^. The molecular identity of S2R remains obscure. A recent study suggests that S2R is the progesterone receptor membrane component 1 (PGRMC1)^4^. However, this conclusion remains to be verified by other independent studies.

The S1R is expressed in many cell types including neurons in the retina and brain. The functional importance of S1R in the nervous system is manifested by the link of its gene defects or reduced protein levels with fronto-temporal lobar degeneration-motor neuron disease^5^, amyotrophic lateral sclerosis (ALS)^6^,^7^, Alzheimer’s disease (AD)^8^,^9^,^10^, Parkinson’s disease (PD)^11^, and Schizophrenia^12^ in human patients. A neuroprotective role of the S1R has been reported in animal models of neurodegenerative diseases including ALS^13^,^14^, AD^15^, PD^16^, and Huntington’s disease^17^. In addition, S1R has also been shown to play an important role in cognition^18^, drug addiction^19^, locomotor activity^20^, and pain^21^. The functional mechanism of S1R is linked to its chaperoning activity for, or functional interactions with ion channels as well as G-protein coupled receptors^22^. Alternatively, S1R has also been shown to regulate Ca^2+^ homeostasis and alleviate unfolded protein responses as well as suppress reactive oxygen species (ROS)^1^.

While numerous studies suggest that S1R is involved in multiple signaling pathways, conflicting evidence exists, and the exact molecular mechanism of S1R-mediated neuroprotection remains unclear. Subcellular localization of S1R provides important information for its functional mechanisms. For example, studies show that S1R resides in the interface of ER and mitochondria^2^,^15^. When stimulated by an agonist or in response to ER stress, S1R is able to translocate to the plasma membrane region to interact with ion channels^3^. In previous studies, immunocyto (or histo)-chemistry was predominantly applied to investigate S1R subcellular distribution. However, this approach provides very limited resolution, resulting in ambiguity in S1R subcellular localization, *e.g.* in the plasma membrane or in subsurface ER cisternae; in the ER or in the nuclear envelope. Thus, in order to determine S1R subcellular localization precisely and unambiguously, a high-resolution method such as electron microscopy (EM) must be applied. Our previous study using EM (~1 nm resolution) identified that in motoneurons S1R localizes in subsurface ER cisternae rather than in the plasma membrane^23^.

In contrast to studies on S1R in other neuronal systems, there are only a small number of reports available on S1R in the retina. These studies generally point to a protective role of S1R. Through a pharmacological approach Bucolo *et al.* observed a protective effect of S1R agonists in reducing retinal damage by using gross retina samples^24^. The Smith group and Yorio group found that S1R agonists protect primary retinal ganglion cells as well as a RGC-5 neuronal cell line against stress-induced cell death, through an antioxidant mechanism^25^ or inhibition of Ca^2^^+^ channel activity^26^, respectively. Our group found that compared to wild type mice, there was a significantly smaller number of surviving cells in the retinal ganglion cell layer in S1R knockout mice after optic nerve crush^27^. Late-onset ganglion cell loss was also reported in aged S1R knockout mice^28^.

There has been a lack of studies on the function of S1R specifically in photoreceptors, likely due to the difficulty to isolate and maintain primary photoreceptor cells. Being the site of initiation of visual signaling, photoreceptors are critically important for normal vision. Dysfunction or death of photoreceptors leads to visual impairment or permanent loss of vision. Moreover, photoreceptor cells are situated in a harsh environment with high levels of ROS that is produced due to active metabolism and light absorption in these cells^29^. It is thus important to investigate whether S1R resides in photoreceptors and plays a protective role. Our previous study using immuno-histochemistry (IHC) indicates that S1R exists in the mouse photoreceptor layer although its subcellular distribution remains unsolved due to the low resolution of this technique^27^. An independent study using rats did not show positive S1R immunostaining in the photoreceptor layer^30^. Therefore, this discrepancy on the presence of S1R in photoreceptors as well as the ambiguity of S1R subcellular distribution underscore the necessity of investigating S1R subcellular distribution in the retina in greater details using EM. An EM study on S1R subcellular distribution in the retina has not been reported. Moreover, the temporal expression of S1R during retinal development remains unknown.

In this study, we first detected S1R using IHC on mouse retina sections of different postnatal days to gain an overview of temporal and spatial distribution of this receptor, which showed its appearance in early developmental stage and distribution throughout the neuronal layers. We then combined immuno-EM with IHC to investigate subcellular localization of S1R in retinal neurons. We found that S1R was predominantly localized in the nuclear envelope of photoreceptor, bipolar, and ganglion neurons but not in the plasma membrane. The mechanistic implications of our findings will be discussed.

## Results

### The expression of S1R in the retina occurs in early developmental stages

**Figure 1:**
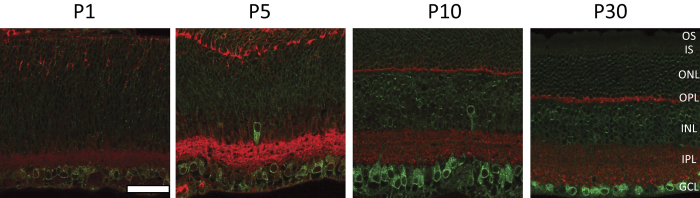
Immunostaining of S1R on mouse retinal sections at different postnatal developmental stages. Green, S1R; red, synaptophysin. OS, outer segment; IS, inner segment; ONL, outer nuclear layer; OPL, outer plexiform layer; INL, inner nuclear layer; IPL, inner plexiform layer; GCL, ganglion cell layer. Scale = 50 μm.

The expression of the S1R in the course of the retinal development has not been previously reported. For an overall survey of temporal and spatial expression of S1R in the retina we performed IHC to detect S1R on mouse retinal sections collected on postnatal days 1, 5, 10, and 30 (Fig. 1). We used an anti-S1R antibody that was in-house produced against the full-length S1R recombinant protein^31^. This antibody produces highly specific IHC in our previous studies, as manifested by negative staining on retinal sections and other samples from S1R knockout mice^23^,^27^. In order to label the inner and outer plexiform layers we immuno-stained synaptophysin, a commonly used presynaptic marker^23^, and the retinal cell layers then became readily distinguishable. We found that on postnatal day 1 (P1) S1R was already expressed in the retina with a higher level in the ganglion cell layer. The expression of S1R increased continuously through P10, a time point when mature retinal layers started to form, as evidenced by compact inner and outer plexiform layers in contrast to the loose retina structure at P1 and P5. The S1R only slightly increased in the adult retina (P30) compared to P10. We also determined S1R expression in E16 embryonic retinae (Supplemental Figure S1), which showed only a minor amount of staining. These data indicate that S1R expression occurs early during the development of the retina and approaches the highest level on P10.

### The S1R is found in the photoreceptor synaptic terminal of the bovine retina but not that of the mouse, monkey, or human retina

**Figure 2:**
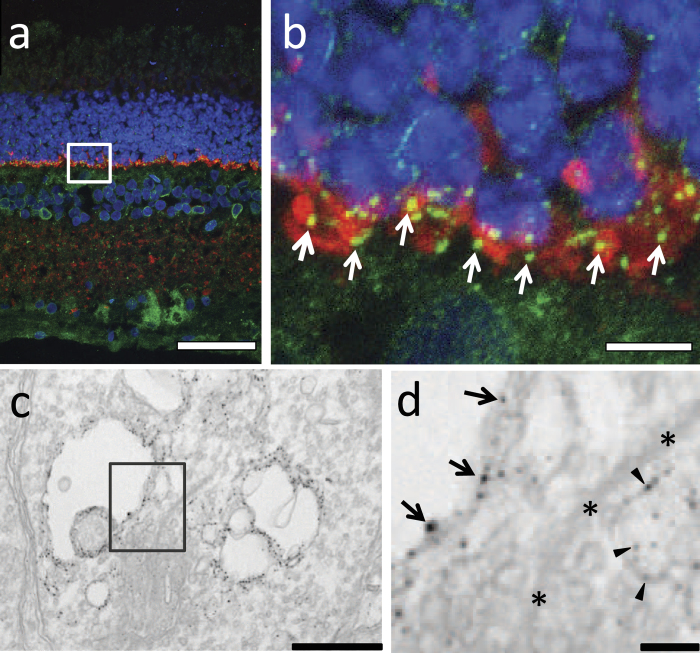
Presence of S1R in the bovine photoreceptor synaptic terminal. (**a**), Confocal image of bovine retina immunostained for S1R (green) and a presynaptic marker, synaptophysin (red). Note positive S1R staining in the OPL. (**b**), Magnifed image of the boxed area in (**a**). Arrows indicate localization of S1R in synaptophysin-labeled synaptic terminals. (**c**), Electron microscopy image showing S1R in the photoreceptor synaptic terminal. Note the characteristic ribbon and vesicles in the boxed area. (**d**), Magnified image of the boxed area in (**c**). Arrows indicate S1R in presynaptic invagination. Arrowheads point to S1R localization in large vesicles. *, synaptic ribbon. Scales: (**a**), 50 μm; (**b**), 5 μm; (**c**), 0.5 μm; (**d**), 0.1 μm.

Mice are nocturnal animals. In order to gain a general view of S1R distribution in adult retinae across other mammalian species including diurnal animals, we surveyed the S1R expression in pig, cow, monkey, and human retinal sections using IHC (Fig. 2; Supplemental Figures S2-S4). S1R distribution in the retina of these species has not been reported previously. Similar to mouse, the retinal sections of all the foregoing species showed S1R distribution throughout the retinal layers but more abundant in the ganglion cell layer. However, one surprising finding was the presence of S1R in the synaptophysin-marked photoreceptor presynaptic region of the bovine retina (Fig. 2a,b). We then performed immuno-EM experiments and confirmed that S1R was indeed localized in the presynaptic region although a possible presence of S1R in the postsynaptic region could not be completely ruled out (Fig. 2c,d). Interestingly, a localization of S1R in the photoreceptor synaptic terminal was observed only in bovine retinae but not in other mammalian species that we have examined.

### The S1R resides in the nuclear envelope of the mouse photoreceptor cell but not in the inner and outer segments

**Figure 3:**
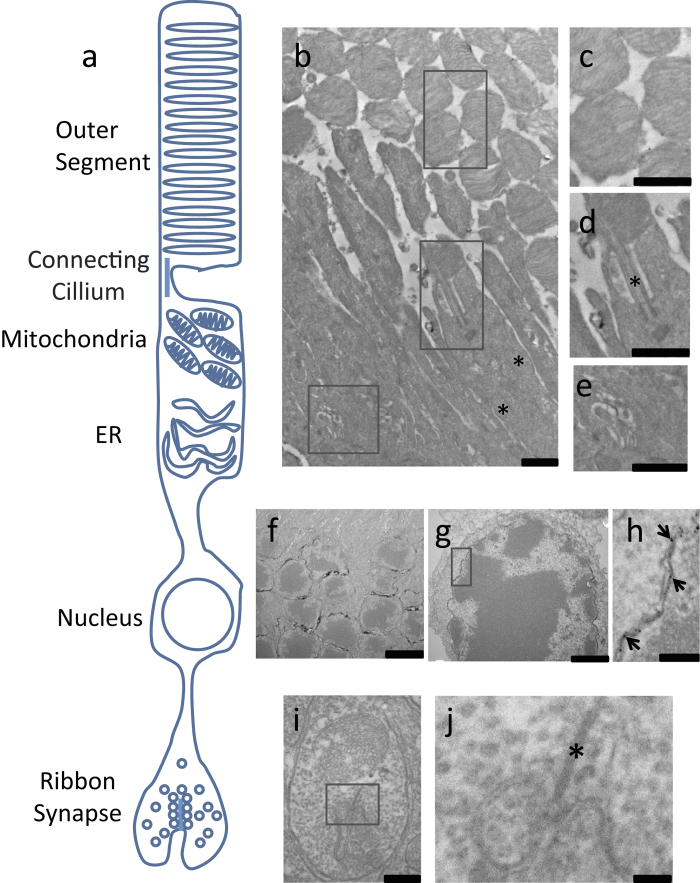
Electron microscopy images showing S1R distribution in the mouse photoreceptor subcellular compartments. (**a**), Schematic of the compartments in the photoreceptor. (**b**), Ultrastructure of outer and inner segment. Asterisks label mitochondria. (**c**)–(**e**), Magnified images of the boxed areas in (**b**), showing the outer segment containing membrane discs, the connecting cilium (asterisk), and the inner segment (including ER), respectively. (**f**)–(**h**), Localization of S1R in the nuclear envelope. (**f**), nuclear region of several photoreceptor cells; (**g**), nuclear envelope of a single cell; (**h**), magnified box area in (**g**) showing S1R localization in the outer and inner membranes of the nuclear envelope (pointed to by arrows). (**i**) and (**j**), Photoreceptor synaptic terminal. The image in (**j**) is a magnified box area in (i) revealing the characteristic ribbon (asterisks) and vesicles. Scales: (**b**)–(**e**) and (**g**), 1 μm; (**f**), 3 μm; (**h**), 0.2 μm; (**i**), 0.5 μm; (**j**), 0.1 μm.

Previous studies by Ola *et al.*^32^ and our own group^27^ have shown the presence of the S1R in the mouse photoreceptor layer, but its exact subcellular localizations remain unclear due to low resolution of the IHC method. Moreover, negative IHC staining of S1R in the rat retina^30^ raised a controversy with regard to the existence of S1R in photoreceptor cells. We thus determined subcellular S1R localizations in mouse photoreceptor cells using immuno-EM. A rod or cone photoreceptor cell is composed of four highly specialized compartments: outer segment (OS), inner segment (IS), nuclear region, and synaptic terminal (Fig. 3a). We prepared sections in such a way that the whole photoreceptor cell was cut in a slanted angle so that all subcellular compartments could be imaged. We did not find S1R-positive electron-dense EM signal in the OS, which features tightly packed membrane discs. We did not see S1R-positive signal in the IS either, which is characterized with packed mitochondria and ER (Fig. 3b). Interestingly however, we observed a clear presence of S1R in the nuclear region, which is not in the ER or plasma membrane but in the nuclear envelope membranes (Fig. 3f–h). In contrast to bovine photoreceptors, no S1R was detected in mouse synaptic terminals (Fig. 3i,j).

### The S1R resides predominantly in the nuclear envelope of bipolar cells and ganglion cells but not in ganglion cell plasma membrane and dendrites

**Figure 4:**
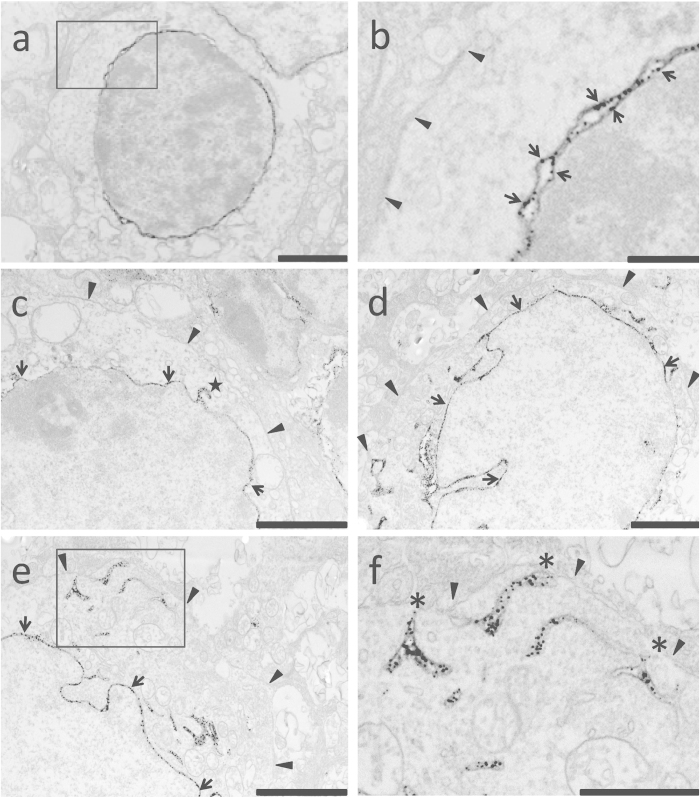
Subcelluar localization of S1R in bipolar and ganglion cells of the mouse retina. (**a**)–(**c**), Bipolar cells. (**b**) shows magnification of the boxed area in (**a**). Arrows point to S1R immunolabeling in the inner and outer membranes of the nuclear envelope. Arrowheads mark the plasma membrane. (**c**) shows S1R localization in the ER membrane (star) connected to the nuclear envelope (arrows). (**d**)–(**f**), Ganglion cells. (**d**) shows predominant S1R localization in the nuclear envelope (arrows) but not in the plasma membrane (arrow heads). (**e**) highlights the presence of S1R in the ER (boxed area). (**f**) shows the magnification of the boxed area in (**e**), revealing S1R localization in the ER cisternae (asterisks) that are adjacent to the plasma membrane (arrow heads). Scales: (**a**), (**c**)–(**e**), 2 μm; (**f**), 1 μm; (**b**), 0.25 μm.

Our EM imaging experiments indicate that in bipolar cells S1R resides predominantly in the nuclear envelope, in both the inner membrane and outer membrane (Fig. 4a–c). We could not detect S1R in the plasma membrane, although occasionally a minor amount of S1R was found in the ER membrane that is connected to the nuclear envelope (Fig. 4c). Similarly, in ganglion cells we found intense S1R-positive labeling in nuclear envelope membranes (Fig. 4d). We also observed somewhat strong S1R labeling in the ER, distal or proximal to the nuclear envelope (Fig. 4e,f), and possibly also in lipid droplets (data not shown). By screening through multiple sections we did not detect S1R in the plasma membrane, although we were not able to rule out the possibility that some minor amount of S1R in the plasma membrane could not be detected due to the low background of the EM method. Instead, we found S1R in the subsurface ER cisternae which are in close proximity to the plasma membrane (Fig. 4e,f). The specificity of the immuno-detection of S1R is supported by the absence of EM signal in the control without using the primary antibody (Supplemental Figure S5).

**Figure 5:**
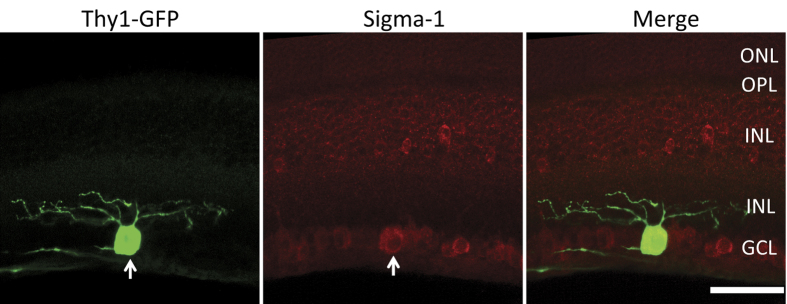
Presence of S1R in the soma and its absence in the dendrites of mouse retinal ganglion cells. A retinal ganglion cell (green) is illuminated by Thy-1 driven GFP expression in the Thy-1/GFP mouse retina. Immunostaining of S1R is shown in red. Arrow points to the GFP positive ganglion cell. Note that while S1R positive staining is seen in the soma it is not evidently detected in the dendrites. Scale = 50 μm.

The retinal ganglion neuron features many dendrites formed by extended plasma membrane that accommodates functionally important ion channels. A number of studies have demonstrated S1R modulation of ion channels^22^. We therefore further determined whether S1R resides in the ganglion dendrites. Since EM images reveal only a single plane of the retina sample in which the thin structure of a dendrite is often severed by sectioning, we used IHC instead to image ganglion dendrites. We utilized a thy1-GFP transgenic mouse line that expresses GFP specifically in a small portion of retinal ganglion cells. As shown in Fig. 5, GFP fluorescence illuminated the fine structures of ganglion dendrites on a retinal section. Superimposing the GFP fluorescence and S1R immunofluorescence showed the presence of S1R in the soma but not in the dendrites.

## Discussion

Growing literature suggests that S1R is a potential target for interventions to treat neurodegenerative diseases including retinal degeneration, although the molecular underpinning for its neuroprotective effect remains underexplored^22^. There has been a knowledge gap with regard to the precise subcellular localization of S1R in retinal neurons. This information is key to understanding the S1R functional mechanisms, especially because the retina is composed of intricately wired neurons, each with multiple subcellular compartments. In this study, using immuno-EM we were able to overcome the ambiguity arising from low resolution methods in previous reports and to delineate S1R subcellular localizations in retinal neurons. We found unambiguous presence of S1R in the mouse photoreceptor nuclear envelope but not in other subcellular compartments, and predominant S1R localization in the nuclear envelope of bipolar cells and ganglion cells. While EM imaging did not show S1R in the plasma membrane in all three types of retinal neurons, we found S1R in ganglion cell subsurface ER cisternae in the plasma membrane region. As discussed below, our results implicate S1R functional mechanisms distinct from previous proposals.

Photoreceptor cell death leads to retinitis pigmentosa and age-related macular degeneration, both major diseases causing blindness in the developed world^29^. It is thus imperative to explore whether S1R could potentially serve as a neuroprotective target in photoreceptor cells. However, there remains a controversy with regard to the existence of S1R in photoreceptors. The distribution of S1R in the photoreceptor subcellular compartments has not been unequivocally addressed. While previous studies by Ola *et al.*^32^ as well as our group^27^ showed positive immunostaining of S1R in the mouse photoreceptor layer, a recent study could not detect S1R in photoreceptors of the rat retina by IHC^30^. Here our EM data revealed the clear presence of S1R in the photoreceptor nuclear membrane. Possible reasons for this discrepancy may include a difference between mouse and rat retinae, different sources of antibodies for IHC, and the fact that S1R abundance in the photoreceptor cell is relatively low. Furthermore, our EM approach with a 1-nm resolution (Figs. 3,4) showed unequivocal S1R localization in the nuclear envelope but not plasma membrane, consistent with previous IHC studies showing “ring” like S1R staining around the nucleus in the outer nuclear layer^27^.

It is interesting to note that whereas IHC showed low levels of S1R in the inner segment, with EM we could not detect S1R in this compartment of photoreceptors. One possible explanation is that EM detects only a single plane and thus could not effectively visualize a sparse S1R presence. In contrast, IHC confocal microscopy detects certain depth and hence the sum of immunofluorescence from multiple planes. Another possibility is that S1R is synthesized in the inner segment, as evidenced by abundant S1R gene transcripts^32^, and then is translocated to the nuclear envelope, resulting in its accumulation consistent with its long turnover time^33^. Alternatively, the S1R presence detected by IHC in the inner segment may represent non-specific background fluorescence. Rod and cone photoreceptor cells are highly specialized for their phototransduction function. All the cellular machineries including ER and mitochondria are localized in the inner segment. All the outer segment resident proteins are synthesized in the inner segment and then transported to their destination — a highly energy consuming process^34^. Moreover, outer segments renew ~10% every day. As such, photoreceptors undergo active metabolism in mitochondria and protein synthesis in the ER; defects in these processes could lead to photoreceptor degeneration^29^. It has been reported that in various cell types including neurons S1R resides in the mitochondria-associated ER membrane (MAM) and protects cell survival during ER or mitochondrial stress^15^,^22^. However, we were not able to detect prominent S1R presence in the ER and mitochondria. Our finding is somewhat surprising and seemingly discordant with the general characterization of S1R in other cell types. While this discrepancy could be rationalized by the highly specialized compartmentalization in photoreceptors, it also underscores the necessity to investigate whether S1R plays a protective role also in photoreceptor cells. Interestingly, the expression of S1R on P10 approximated the adult level, consistent with the time of eye-opening that is accompanied by light-evoked retinal stresses. In future studies, the predominant localization of S1R in the nuclear envelope should be taken into consideration, which may imply a unique mechanism for the function of S1R in photoreceptor cells.

Previous IHC studies^27^,^32^ and our EM data showed the presence of S1R in postsynaptic bipolar cells, although the localization of S1R in the photoreceptor presynaptic terminal has never been specifically addressed. By immunostaining S1R on retinal sections from several mammalian species, we found a S1R presence in presynaptic terminals only in the bovine retina, which was confirmed by EM. However, we cannot completely rule out the possibility of minor amount of S1R present in the photoreceptor synaptic terminal of other species. The photoreceptor synaptic terminal houses the ribbon structure enriched with neurotransmitter vesicles and various ion channels on the plasma membrane that execute visual signal transmission from photoreceptors to secondary neurons^35^. Thus the unique localization of S1R in bovine presynaptic terminals raises an interesting question as to whether S1R influences visual signaling by modulating retinal synaptic activities.

Ganglion cell death occurs in the later stage of glaucoma — another major disease in Western countries^27^. Studies on S1R in the retina show its prominent abundance in ganglion cells^27^,^30^,^32^. Likely because of its abundance, several studies have investigated a protective role of S1R in ganglion cells *in vitro* and *in vivo*. One of the proposed mechanisms for the protective effect of S1R against ganglion cell death is the inhibition of ion channel activities by S1R. In a RGC-5 neuronal cell line as well as in purified primary retinal ganglion cells, activation of S1R was shown to effectively inhibit the L-type voltage-gated calcium channel and hence calcium influx, which was proposed to play a role in protection of ganglion cells^26^,^36^,^37^. Direct S1R/channel interaction was detected by co-immunoprecipitation and co-localization^26^. In another independent study, activation of S1R suppressed NMDA receptor in both on and off ganglion cells, which was proposed to protect the cells from harmful overstimulation of the channel^38^. Our finding here is provocatively interesting because EM imaging did not identify S1R in the plasma membrane of ganglion cells in the mouse retina, raising a question about the accessibility of S1R to the foregoing mentioned channels in the plasma membrane. To reconcile our EM imaging results with previous studies, we propose possible explanations as follows. 1. Due to the low background nature of the EM approach, sparsely distributed S1R in the plasma membrane may not be detectable. On the other hand, even a small number of S1R/channel interactions could be measured thanks to the signal amplification through an electrophysiology approach (*e.g.* patch clamp). 2. In the presence of an agonist S1R may translocate from ER to the plasma membrane. This scenario had been proposed earlier although a clear mechanism has yet to be unraveled^1^. Recent studies showed some biochemical evidence of increased S1R presence in the plasma membrane after agonist stimulation^19^. Future studies are warranted to monitor agonist-induced S1R appearance in the ganglion cell plasma membrane using EM. 3. Since S1R is known to regulate cellular calcium homeostasis by chaperoning the IP3 receptor in the ER^2^, it may influence channel activities in the plasma membrane indirectly through the change of intracellular calcium levels^38^. 4. Alternatively, S1R in the subsurface ER cisternae may be in sufficient proximity to the channels in the plasma membrane for direct interaction. Indeed, through EM imaging we have previously shown that in motoneurons the subsurface ER cisternae, where abundant S1Rs are detected, are in close proximity (<10 nm) to the plasma membrane which supports a possibility for direct S1R/channel interactions^23^.

It is worth noting that in all three retinal neurons we have investigated, *i.e.* ganglion cells, bipolar cells, and photoreceptor cells, S1R was localized predominantly in the nuclear envelope. In support of our finding, Jiang *et al.*^39^ observed strong co-localization of S1R and the nuclear membrane marker lamin-A in cultured Mueller cells; another early report also showed EM evidence of S1R localization in nuclear membranes of human THP1 cells^40^. There is an interesting contrast between the IHC images that show S1R staining not only in the peri-nuclear area but also throughout the cytosol and the EM images that show majority of S1R in the nuclear envelope and only a portion in the ER. The simplest explanation is that confocal imaging of IHC detects S1Rs at various locations on multiple planes of the retina thus presenting overlaid images, whereas EM imaging is able to reveal relative abundance of S1R in subcellular organelles on the same plane. Likely for this reason, a predominant S1R abundance in the nuclear envelope has been previously overlooked. The functional significance of the S1R localization in the nuclear envelope is not known. There is a recent report showing S1R in the nucleus^41^, however the finding hasn’t been confirmed by other independent studies. It remains an intriguing question as to whether S1R participates in the regulation of gene expression or protein trafficking between the nucleus and the cytoplasm.

In summary, this study is the first to determine precise subcellular localizations of the S1R in the retinal neurons using EM. S1R is shown not to localize on the ER or mitochondria of photoreceptors, implicating a functional mechanism possibly different from that previously determined in other cell types. A predominant S1R presence in the nuclear envelope of retinal neurons was under-appreciated in previous studies using IHC, and may suggest yet-to-be identified functions of S1R associated with nuclear activities. The localization of S1R in the subsurface ER cisternae may provide sufficient proximity for the S1R interactions with ion channels in the plasma membrane. Thus, our study provides mechanistic insights with general significance for better understanding of the S1R-mediated regulations in neurons.

## Materials and methods

### Ethics Statement

All animal procedures conform to the Guide for the Care and Use of Laboratory Animals (NIH publication No. 85-23, 1996 revision) and in compliance with the ARVO Statement for the Use of Animals in Ophthalmic and Vision Research. Animal protocols were approved by the Institutional Animal Care and Use Committee at the University of Wisconsin. All surgeries were performed under isoflurane anesthesia (through inhaling, flow rate 2 ml/min), and all efforts were made to minimize suffering. Animals were euthanized in a chamber gradually filled with CO_2_.

### Animals

C57BL/6 mice and Thy1-GFP mice (stock# 007788) were purchased from the Jackson Laboratory. Animals were maintained on a 4% fat diet (8604 M/R, Harkland Teklad, Madison, WI) and subjected to standard light cycles (12 h/12 h light/dark).

### Immunohistochemistry and confocal microscopy

Following euthanasia of the mice, eye balls were enucleated immediately and dissected. The eyecups were fixed in 4% paraformaldehyde for 7 h, and then cryoprotected in 30% sucrose in PBS for another 14 h, all at 4 °C. Cryosections of 10 μm each were cut from the eyecups frozen in the optimum cutting temperature (O.C.T.) embedding medium (Sakura Finetek 4583, Sakura Finetek USA, Inc., Torrance, CA), and used for immunostaining following our method described previously^42^ with minor modifications. Briefly, retinal sections were permeabilized with 1% Triton X-100 in PBS for 20 min, blocked with 10% normal goat serum (Cat#71–00–27; Kirkegaard & Perry Laboratories, Gaithersburg, MD) for 2 h at room temperature, and then incubated with purified rabbit anti-sigma-1 receptor antibody^31^ (1/150 dilution) and mouse monoclonal anti-synaptophysin (Cat. #MAB368, 1/500 dilution; Chemicon, Los Angeles, CA) overnight at 4 °C. After rinsing the sections 3×, secondary antibodies (Alexa-488 conjugated goat-anti-rabbit and Alexa-594-conjugated goat-anti-mouse) at 2 μg/ml was applied at room temperature for 2 h. Sections were then rinsed 3×, counterstained with 4’,6-diamidino-2-phenylindole (DAPI) for 5 min, and then mounted in the Prolong Gold mounting medium (Invitrogen, Carlsbad, CA) and cover-slipped. The slides were left in the dark overnight and then sealed using clear nail polish (Electron Microscopy Sciences, Hatfield, PA). Images were then taken with a Nikon A1R laser confocal microscope (Nikon, Tokyo, Japan) supplied with a green 488 nm Argon laser and a red 561 nm DPSS laser through an Apo60X VC oil-immersion objective with NIS elements software^23^. Z-stacks were collected at 0.5 μm each, for a total thickness of 10 μm. Sequentially collected images were stacked together using the ImageJ program.

The porcine, bovine, monkey, and human retina sections were prepared similarly. The porcine and bovine eyeballs were obtained from the Black Earth Meats slaughter house in Cross Plain, WI, USA. The monkey retinal sections were from the Primate Research Center, University of Wisconsin, Madison, WI, USA. The human eyeball was procured from the Minnesota Lion Eye Bank.

### Immunoelectron microscopy

We performed immuno-EM experiments following our published methods^23^. Mice were intracardially perfused with 4% PFA and 0.2% glutaraldehyde in 0.1 M phosphate buffer. Eyeballs were dissected and post-fixed in the same fixative overnight. Retinas were dissected and 60 μm thick sections were cut using a Leica VT 100S vibratome. Sections were quenched in 1% sodium borohydrate for 30 min, rinsed with PBS and permeabilized in 0.05% Triton X-100 for 15 min, and then blocked in normal goat serum for 1 h. Sections were incubated with primary anti-sigma-1 receptor antibody (1/150 dilution in PBS) for 48 h at 4 °C. Immunostaining was further revealed with ABC peroxidase kit (Vector Laboratories, Burlingame, CA, USA) and a mixture of 0.02% diaminobenzidine and 0.01% H_2_O_2_ in 50 mM Tris, pH 7.6 for 10 min. The sections were then rinsed and post-fixed with 2% glutaraldehyde for 30 min followed by washing 3× in 100 mM Tris-Maleic Acid. Electron-dense polymer of diaminobenzidine was further intensified by a mixture of 0.52% hexamethyltetramine, 0.04% silver nitrate, and 0.04% sodium tetraborate all in 100 mM Tris-Maleic Acid buffer pH 7.4 for 10 min at 60 °C in the dark. Then the sections were rinsed in nanopure H_2_O and 0.01 M PBS and placed in 0.05% solution of gold chloride for 5 min. To wash away unbound silver particles samples were first treated with 3% sodium thiosulfate for 2 min, and then washed 3× in water. The samples were then post-fixed with 1.5% osmium tetroxide for 1 h, rinsed and stained en block with 1% uranyl acetate and dehydrated in graded series of ethanol, washed twice with propylene oxide (5 min each time). Samples were further infiltrated in Epon resin/propylene oxide (1:1 ratio), and then in pure Epon, and finally polymerized between two teflon coated glass slides. Thin sections of 70 nm were cut using a Leica EM UC7 ultramicrotome, counterstained in 1% uranyl acetate and viewed and imaged with Phillips CM120 STEM electron microscope.

## Additional Information

**How to cite this article**: Mavlyutov, T. A. *et al.* Subcellular Localization of the Sigma-1 Receptor in Retinal Neurons – an Electron Microscopy Study. *Sci. Rep.* **5**, 10689; doi: 10.1038/srep10689 (2015).

## Supplementary Material

Supplementary Information
