# Supplementary material for: Subcellular Localization of the Sigma-1 Receptor in Retinal Neurons — an Electron Microscopy Study
Source: Sci Rep. 2015 Jun 2;5:10689. doi: 10.1038/srep10689 (PMC4649997; doi:10.1038/srep10689)
Supplement: Supplementary Information [file srep10689-s1.pdf]

Supplementary Figures

Subcellular Localization of the Sigma-1 Receptor in Retinal Neurons — an Electron Microscopy Study

Timur A. Mavlyutov[1,2], Miles Epstein[3], Lian-Wang Guo[1,2,*]

[1]Department of Surgery, [2]McPherson Eye Research Institute, University of Wisconsin School of Medicine and Public Health, 5151 Wisconsin Institute for Medical Research, 1111 Highland Ave, Madison, WI 53705, USA

[3]Department of Neuroscience, University of Wisconsin School of Medicine and Public Health, 41 Bardeen Medical Laboratory, 470 N Charter Street, Madison, WI 53706, USA

[*]**Corresponding author:**

Lian-Wang Guo, PhD

Department of Surgery
University of Wisconsin School of Medicine and Public Health
5151 Wisconsin Institute for Medical Research
1111 Highland Ave, Madison, WI 53705, USA
Phone: 608 262 6269
Email: guo@surgery.wisc.edu

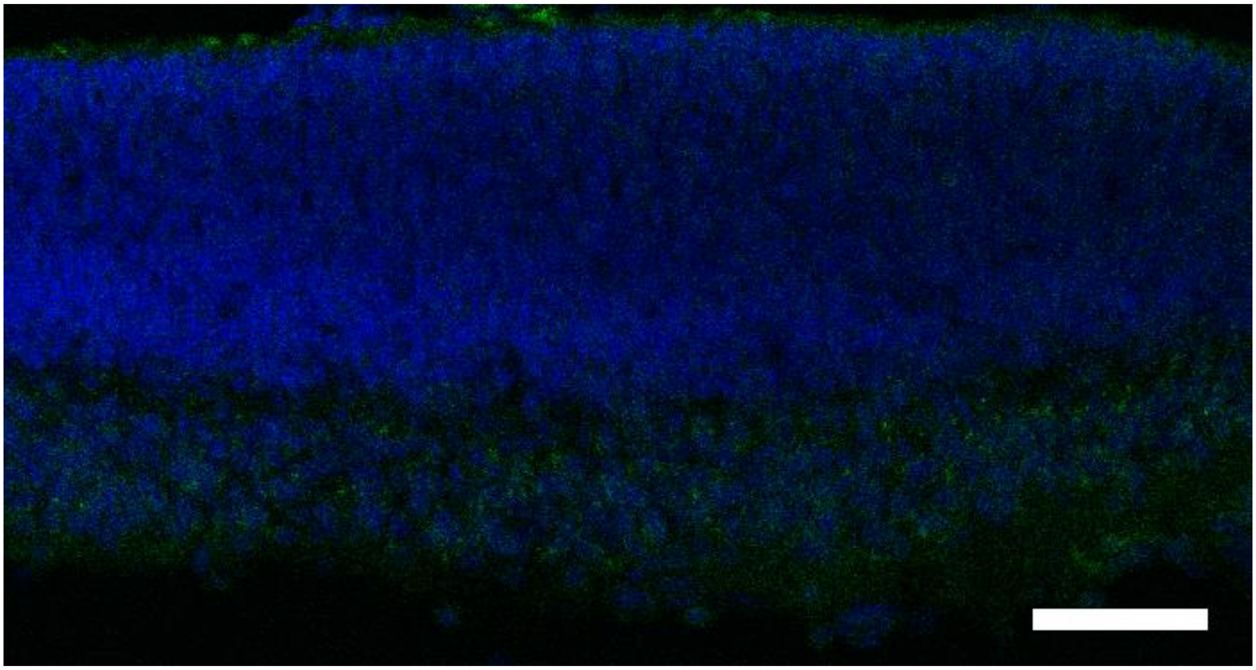

Figure S1.Developing retina of E16 mouse embryo.

At this stage the layers of retina are not distinct. S1R (green) is weakly detected in the bottom layer which will develop into ganglion cells. Nuclei are labeled with DAPI (blue). Scale = 25 µm.

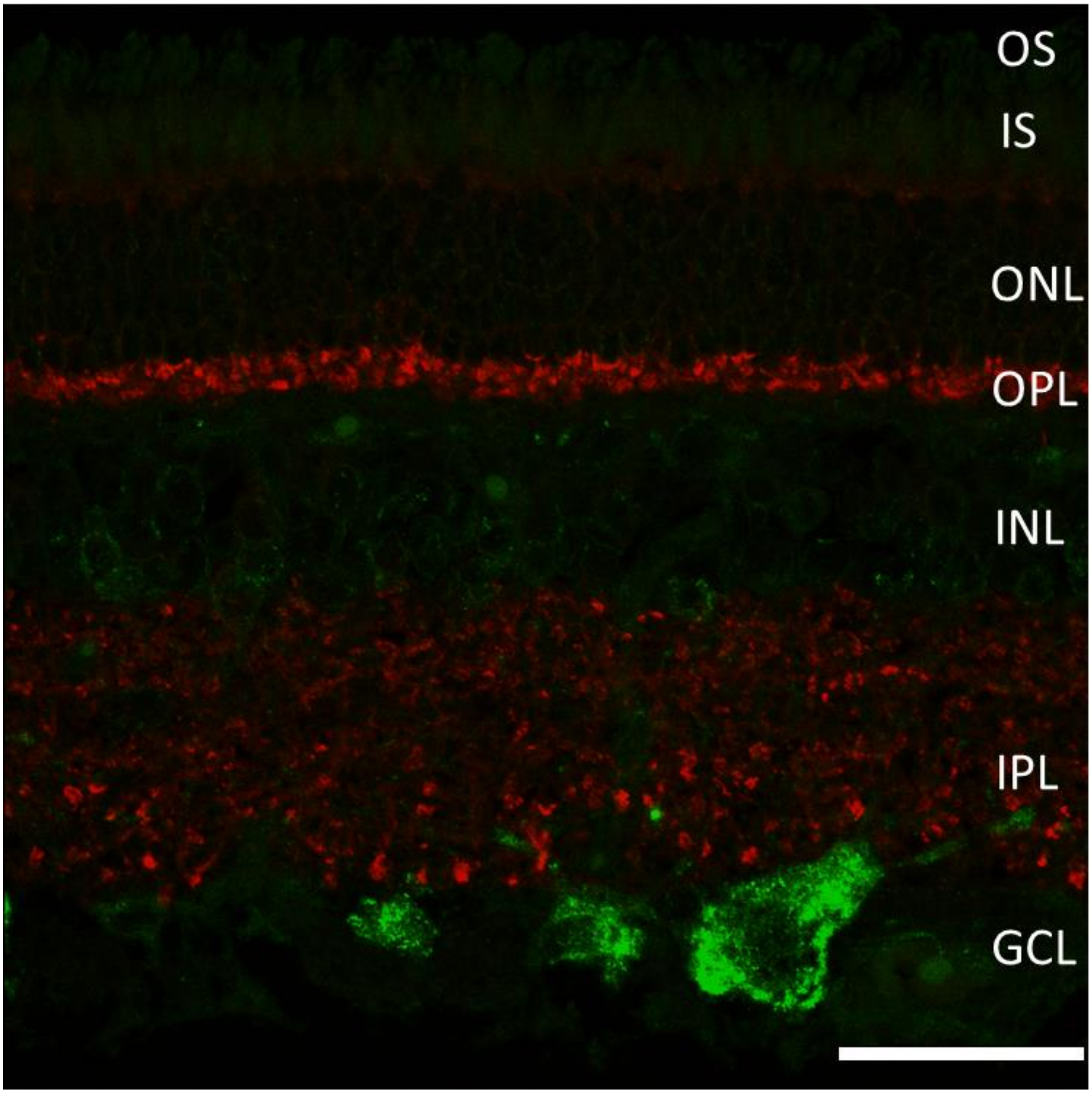

Figure S2. Immunostaining of S1R on the monkey (rhesus macaque) retinal section
Green, S1R; red, synaptophysin; blue, DAPI. Scale = 50 µm.

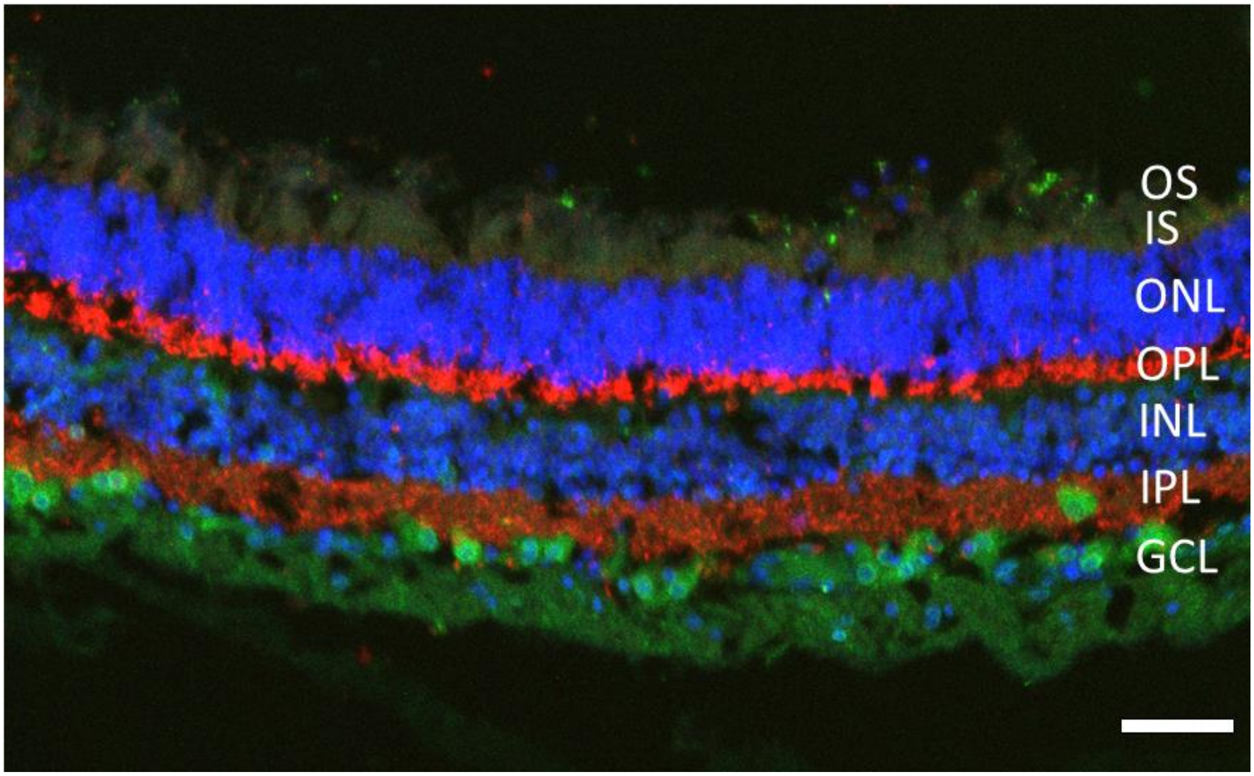

Figure S3. Immunostaining of S1R on the human retinal section

Sections were prepared from a 52 years old male. Green, S1R; red, synaptophysin; blue, DAPI. Scale = 50 µm.

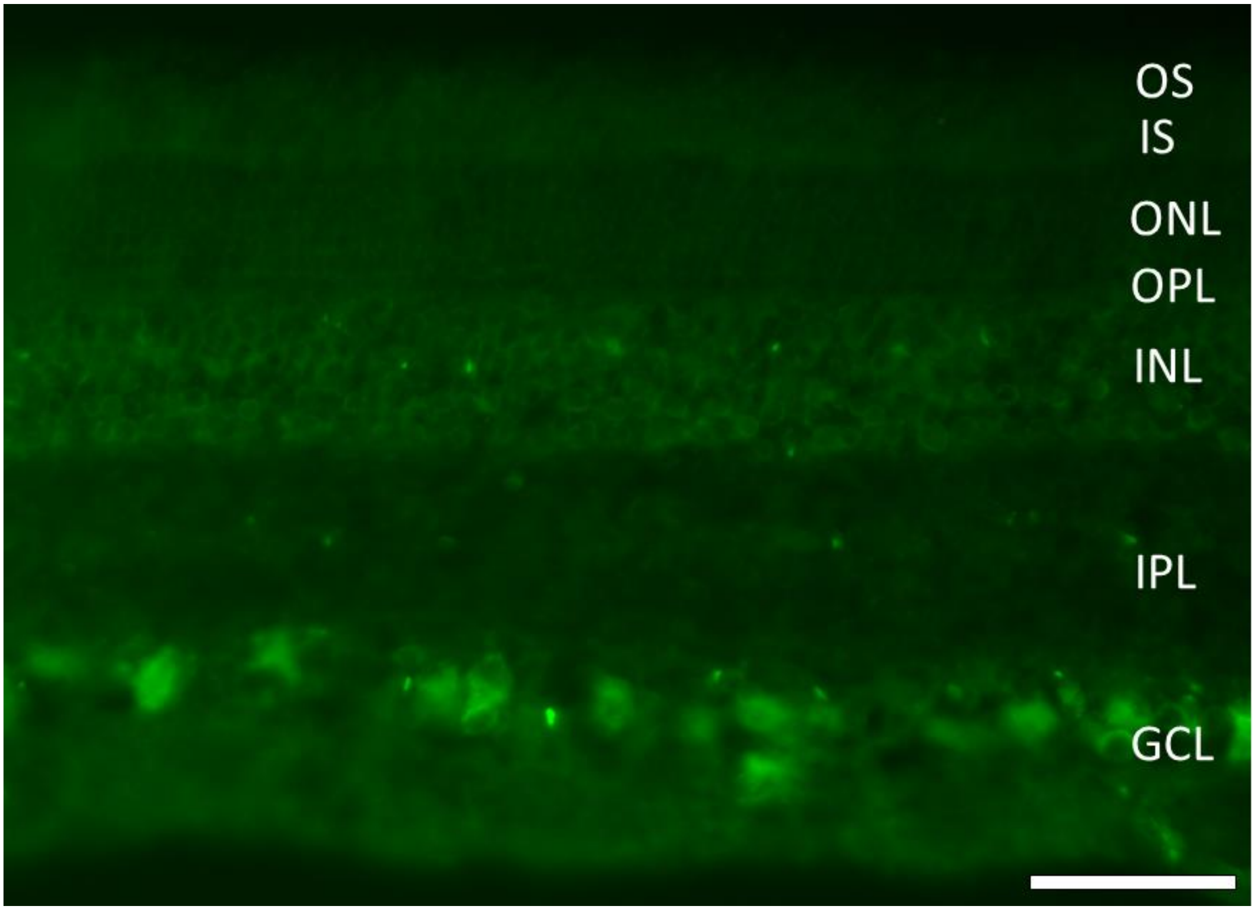

Figure S4. Immunostaining of S1R on the pig retinal section
Sections were prepared from adult pigs. S1R is stained green. Scale = 50 µm.

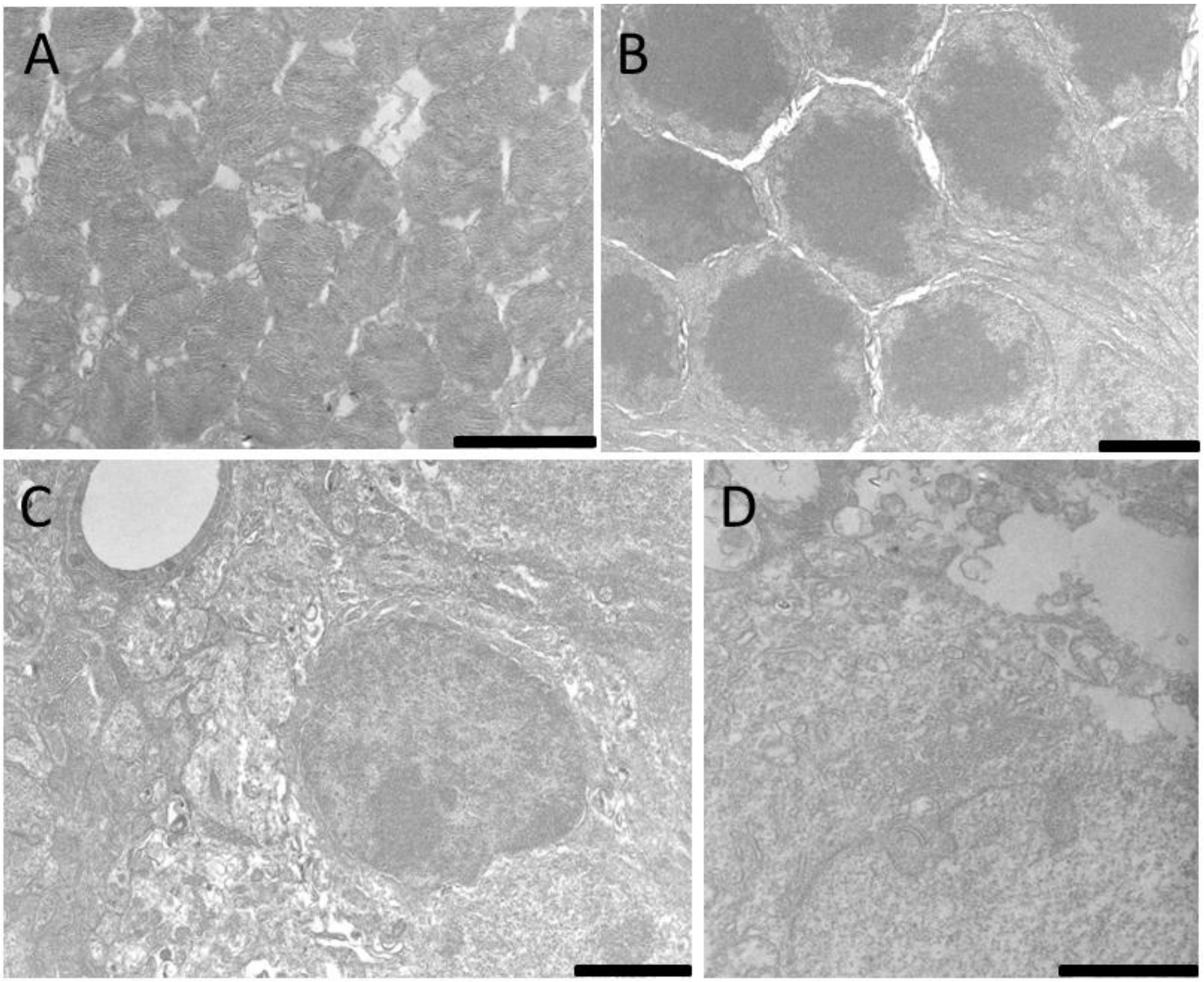

Figure S5. Negative control for immuno-electron microscopic detection of S1R in the mouse retina
The experimental procedures were the same as in Figures 3 and 4 except that the primary anti-S1R antibody was omitted. (a), Outer segment; (b), Nuclei of photoreceptors; (c), Bipolar cell; (d), Ganglion cell. Note the absence of positive S1R immunolabeling. Scale = 2 μm for all.
